# Catheter Ablation of Septal Accessory Pathways in Children: A 12-Year Experience at a Tertiary Care Center

**DOI:** 10.3390/jcdd12040111

**Published:** 2025-03-23

**Authors:** Ozlem Turan, Celal Akdeniz, Volkan Tuzcu

**Affiliations:** 1 Department of Pediatric Cardiology, Antalya Training and Research Hospital, University of Health Sciences, 07100 Antalya, Turkey; 2 Department of Pediatric Cardiology/Electrophysiology, Medipol University, 34214 Istanbul, Turkey; celalakdeniz@yahoo.com (C.A.); vtuzcu@gmail.com (V.T.)

**Keywords:** cryoablation, irrigated-tip radiofrequency ablation, recurrence

## Abstract

**Background**: Septal accessory pathways (APs) are challenging ablation targets. This study aims to contribute to the pediatric literature by presenting our long-term experience of septal AP ablations with limited fluoroscopy. **Methods**: This is a retrospective study of all patients who underwent septal AP ablations from July 2012 to July 2023 at a single center. **Results**: We identified 298 septal AP connections in 291 (11.8 ± 4.9 years) patients. Seventy-nine (27%) cases were diagnosed with supraventricular tachycardia, and 212 (73%) cases were diagnosed with Wolff–Parkinson–White (WPW). The AP locations were posteroseptal (*n* = 159; 54%), anteroseptal (*n* = 86; 30%), and midseptal (*n* = 46; 16%). Of those diagnosed with WPW, 61 (28%) had high-risk AP, and 90 (40%) were adenosine-responsive. Cryoablation was used in 190 (66%), radiofrequency ablation (RFA) was used in 36 (12.5%), and both were used in 62 (21.5%) patients. The overall acute success rate of initial procedures was 89.6% (the acute success rate of cryoablation = 86.6%, and of RFA = 94.1%). No statistically significant difference was observed between cryoablation and RFA (*p* = 0.617). During a mean follow-up of 88.5 ± 33.0 months, the overall recurrence rate was 11.3% (cryoablation vs. RFA; *p* = 0.834), with the highest at the right-posteroseptal location. An irrigated-tip RFA was preferred during redo procedures in 20 (45%) cases. The long-term success rate was 99% when the repeat procedures were considered. No complications were observed. **Conclusions**: Due to the higher recurrence rates in septal AP ablations compared to other locations, repeated procedures might be needed to achieve definitive long-term success. This study indicates that similar acute and long-term success rates can be achieved with cryoablation compared to RFA, with the significant benefit of increased safety.

## 1. Introduction

Septal accessory pathways (APs) are challenging targets, representing 30% of all APs [1]. Ablation of anteroseptal and midseptal APs increases the risk of atrioventricular (AV) block due to their proximity to the normal AV conduction system. However, AV block may also occur in other septal locations. In contrast, the complex anatomy of the posteroseptal space presents a risk of coronary injury [2].

Radiofrequency catheter ablation (RFA) is the preferred treatment for eliminating accessory pathways [3]. However, RFA for septal APs is linked to higher complication rates than other locations [4]. Since 2003, cryoablation has emerged as a safe alternative to RFA for several arrhythmogenic substrates, particularly in the septal region [5,6,7,8]. The pediatric literature indicates that cryoablation has higher recurrence rates compared to RFA. Nevertheless, some benefits of cryothermal energy, such as lesion reversibility, a reduced risk of thrombus formation, and improved catheter stability, make it a favorable option when the AP is near the AV node or bundle of His [6,8,9].

Although many expert consensus reports on the management of AP in the pediatric population estimate an overall acute procedural success rate of 95%, septal AP ablation presents lower acute success rates, with higher recurrence rates when evaluated over a more extended follow-up period [10,11,12]. To contribute to the pediatric literature, the study aims to describe the characteristics of the procedures and the procedural outcomes of septal AP ablations using limited fluoroscopy at a single center. Additionally, we evaluated both initial and repeat procedures in terms of techniques and strategies to gain a better understanding of the factors that may have influenced long-term outcomes.

## 2. Materials and Methods

### 2.1. Patients and Study Design

This single-center, retrospective study reviewed records of patients undergoing electrophysiological studies (EPS) for septal APs at Medipol University, Istanbul, from July 2012 to July 2023. Using FileMaker^®^ Pro 11.0v3 software, we collected data on patient demographics, AP location, arrhythmia substrates, catheter and energy type, imaging modality, transseptal puncture, procedure and fluoroscopy durations, and complications. Initial ablation data were included for repeat procedures. Given the heterogeneity in the patient data, particularly with regard to mapping techniques, catheters, and energy used for ablation, after overall evaluation of procedures, patients were categorized based on AP localizations. The data for the initial and repeat procedures were presented in each group. The study was approved by our ethics committee.

Inclusion criteria specified patients with atrioventricular reciprocating tachycardia (AVRT) substrates due to either manifest or concealed APs. Manifest APs (WPW) were classified as WPW pattern (asymptomatic ECG findings) or WPW syndrome (ECG findings with symptomatic arrhythmias like SVT or atrial fibrillation), while concealed APs presented with SVT.

### 2.2. Electrophysiologic Study

Informed consent was obtained from all guardians. Antiarrhythmic drugs were discontinued at least five half-lives before the procedure. All procedures were conducted under general anesthesia. Catheters were inserted via femoral venous access using the EnSite 3D (St. Jude Medical, Inc., St. Paul, MN, USA) electroanatomic mapping system (EAM). After respiration compensation, a 3D right atrial map was created, marking the His bundle and measuring the His–ventricular (HV) interval.

A comprehensive EPS was performed to identify the arrhythmia substrate and evaluate its characteristics. Adenosine was used to test AP sensitivity by observing for slowed or blocked conduction. For manifest APs, risk was evaluated using SPERRI, APERP, and SPPCL, with values ≤250 ms indicating high risk for sudden cardiac death and 250–300 ms considered borderline [13,14]. The EAM enables precise 3D mapping, offering a detailed understanding of conduction system distribution. Mapping the septal extension across upper, mid, and lower thirds provides critical insights into electrical activity propagation and facilitates precise ablation targeting.

The location of APs was defined as follows [11].

1. Anteroseptal (AS): defined by earliest ventricular activation during anterograde conduction or retrograde atrial activation during pacing in the upper third of the triangle of Koch, with a His bundle potential (≥0.1 mV) at the ablation site.
2. Midseptal (MS): identified when earliest activation occurs in the middle third of the triangle of Koch.
3. Posteroseptal (PS): located near the CS ostium or proximal CS (right-PS). If on the left septal side opposite the right-PS, it is classified as left-PS AP. Ablations conducted through the CS and middle cardiac vein were categorized under a separate heading as “epicardial ablations”.

If the AP was left-sided, the patent foramen ovale was initially explored; otherwise, an anterograde approach via transseptal puncture under fluoroscopy was used. Heparin was administered to maintain an activated clotting time > 200 s.

Mapping was performed using a steerable quadripolar 6F EPS catheter, with re-mapping via an ablation catheter only when necessary. For clear pre-excitation, the earliest ventricular far-field intracardiac signal was mapped during sinus rhythm. In cases of sustained orthodromic AVRT, the earliest atrial near-field intracardiac signal was mapped during tachycardia. The initial ablation target was the presumed AP intracardiac signal, or, if absent, the earliest activation identified via the LAT map of the 3D EAM system. For concealed APs, retrograde mapping during tachycardia and/or V-pace mapping were used. Additionally, a qS pattern in the unipolar electrogram was targeted during sinus rhythm or atrial pacing in manifest APs. Catheter stability, a key challenge at the tricuspid annulus, was improved using a steerable long sheath (Agilis™, Abbott, Chicago, IL, USA), enhancing mapping effectiveness in selected patients.

### 2.3. Catheter Ablation

For AS and MS APs, cryoablation was the first-line energy, using a 6 mm or 8 mm tip Freezor^®^ Xtra catheter (Medtronic Inc., Minneapolis, MN, USA). The 6 mm tip was the first choice for ablation, especially for patients <30 kg, accounting for availability of material. Cryomapping at −30 °C was performed with a 6 mm tip, stopping after 30–45 s if ineffective. With an 8 mm tip, 30 s test applications were used. In manifest atrial pre-excitation, cryomapping was done during sinus rhythm, orthodromic tachycardia, or ventricular pacing to confirm AP block. For concealed APs, it was performed during orthodromic tachycardia or ventricular pacing. Ablation proceeded at −70 °C to −80 °C for 240–360 s upon AP block, followed by insurance lesions for a freeze–thaw–freeze effect.

RFA was preferred when the ablation area was distant from the AV node or if cryoablation failed. Conventional RF energy was delivered using a 7 FR RF Marinr™ Multi-curve Steerable Catheter (Medtronic, Minneapolis, MN, USA) and an IBI generator (St. Jude Medical, Inc., St. Paul, MN, USA) in temperature-controlled mode at 40–50 watts, with a temperature cut-off of 50–60 °C. Irrigated RFA was preferred for patients >30 kg, particularly when the ablation site was far from the AV node or after RF/cryo failure. For right-PS APs, it was prefered for mapping and ablation, especially in recurrences. Irrigated RF catheters (7 FR 4 mm Cool Flex™ and 8 FR 4 mm Flex Ability™; St. Jude Medical) were used with a temperature-controlled generator, starting at 25 W and increasing to 30–35 W, with a resistance of 85–90 Ω and a temperature range of 30–35 °C. Power was adjusted from 15 W to 20–25 W during energy delivery into the coronary sinus. Lesion duration was limited to 60 s, with two consolidation lesions for RFA and irrigated RFA.

### 2.4. Definition of Procedural Endpoints, Complications, and Follow-Up

AV node conduction was monitored during mapping and ablation, with immediate cessation if AV delay occurred. Acute success is defined as the absence of bidirectional AP conduction at baseline and after adenosine administration, with the absence of other arrhythmia substrates following a 30 min waiting period. Patients were monitored for 24 h, with ECG and echocardiography at discharge, 2 weeks, 3 months, and 6 months. Aspirin was given for 6 weeks after left-sided procedures. Mean follow-up was 88.5 ± 33.0 months. Recurrence was defined as reappearance of AP conduction or SVT on ECG, Holter, or event recorder, while long-term success was defined as the absence of WPW or SVT during follow-up.

### 2.5. Statistical Analysis

The data were analyzed using version 21.0 of the Statistical Package for the Social Sciences. Descriptive features were expressed as percentages and mean ± standard deviation or as medians, depending on the distribution of the data as determined by the Kolmogorov–Smirnov test. The Chi-square test or Fisher’s exact test (when Chi-square test assumptions do not hold due to low expected counts), where appropriate, was used to compare the proportions in different groups. Outcome variables were analyzed using logistic regression, and odds ratios (ORs) were calculated where appropriate. Hosmer–Lemeshow goodness-of-fit statistics were used to assess model fit. A *p*-value of less than 0.05 was considered to show a statistically significant result.

## 3. Results

### 3.1. Patient Characteristics

A total of 291 children underwent 336 EPS and 332 ablation procedures, all guided by the 3D EAM system with limited fluoroscopy. The mean age and weight of the patients at first ablation were 11.8 ± 4.9 years and 48.8 ± 27.3 kg, respectively. Congenital heart disease was present in 21 patients. The youngest patient included in the study was a 4-month-old (5 kg) infant diagnosed with ‘tachycardia-induced dilated cardiomyopathy’ that was resistant to multiple antiarrhythmic drugs. Two hundred and twelve (72.9%) were diagnosed with WPW [71 with WPW pattern, 141 with WPW syndrome (140 with SVT and 1 with pre-excited atrial fibrillation)], while 79 cases (27.1%) were diagnosed with SVT (Table 1).

### 3.2. Accessory Pathway and Procedure Characteristics

We identified 298 APs in 291 patients, with 7 (2.4%) having multiple APs, including 3 with Ebstein’s anomaly. Antegrade conduction was observed in 225 (75.5%) APs, 90 (40%) of which were adenosine-sensitive. Among manifest AP cases, 61 (28%) were high-risk (APERP, SPERRI, SPPCL ≤ 250 ms), 77 (34.5%) borderline (251–300 ms), 82 (36.5%) low-risk (≥300 ms), and 5 (1%) unknown. Mean APERP was 294.4 ± 5.3 (Table 2).

The most common septal AP location was PS (159; 54.6%), followed by AS (86; 29.5%) and MS (46; 15.9%). Five (2%) APs exhibited ‘Mahaim-like properties’ with decremental conduction, where increased pacing aggressiveness prolonged the A-delta interval, indicating slowed conduction. Mapping was performed during SVT in 70 (24%) cases, delta mapping in 116 (40%), both in 71 (25%), and V-pacing in 31 (11%) patients.

Cryoablation was used in 190 patients (66%), RFA in 36 (12.5%), and both in 62 (21.5%) (Table 3). Cryoablation involved a 6 mm catheter in 188 patients, an 8 mm in 62, and both in 2. The overall initial acute success rate was 89.6%, with significant difference between MS and PS locations (*p* = 0.028). The acute success rates of cryoablation and RFA were similar (respectively, 86.6% and 94.1%; *p* = 0.617). Predictors of unsuccessful cases were weight (<30 kg) (OR = 1.7; 95% CI: 0.483–6.482), the use of cryotherapy (OR: 0.868; 95% CI: 0.089–8.436), and PS ablation (OR: 0.625; 95% CI: 0.168–2.232). MS ablation (OR: 0.333; 95% CI: 0.050–2.195) had a lower failure probability.

**Figure 1:**
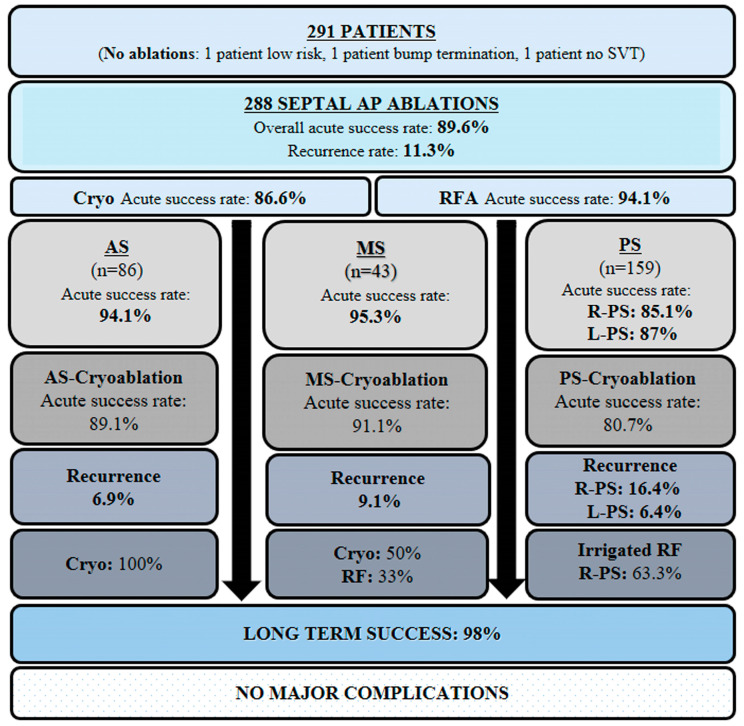
Schematic overview categorizing septal AP ablations and their associated outcomes by anatomical location. AP, accessory pathway; SVT, supraventricular tachycardia; AS, anteroseptal; MS, midseptal; PS, posteroseptal; R-PS, right posteroseptal; L-PS, left posteroseptal; RFA, radiofrequency ablation; Irr, irrigated.

During a mean follow-up of 88.5 ± 33.0 months, the overall recurrence rate was 11.3%, with 33 cases—20 (61%) WPW and 13 (39%) with SVT. Cryoablation had a 12.1% recurrence rate, with no significant difference from RFA (*p* = 0.834). Additionally, no difference was observed between the groups in terms of initial procedure recurrence (AS vs. PS, *p* = 0.083; MS vs. PS, *p* = 0.308; AS vs. MS, *p* = 0.739). The redo procedures were conducted an average of 19.2 months after the initial ablation, with an average of 1.2 procedures per patient. The overall acute success rate of redo procedures was 95.3%. The long-term success rate was 100% for AS, 97.6% for MS, and 98.7% for PS pathways, without any significant difference (AS vs. PS, *p* = 0.861; MS vs. PS, *p* = 0.762; AS vs. MS, *p* = 0.873) (Table 4, Figure 1).

For accessory pathways, age (<8 years) was not signicantly associated with the need for repeat ablation. The variables associated with requiring a repeat procedure were female gender (OR = 2.2; 95% CI: 1.097–4.386), use of cryotherapy (OR = 10.7; 95% CI: 1.008–113.365), and presence of PS AP (OR = 3.2; 95% CI: 1.168–8.972) (Table 5).

In our electrophysiology practice, fluoroscopy is only utilized when a transseptal approach is required or in rare cases to confirm catheter position. Fluoroscopy was used for 39 patients (13.5%), with a mean fluoroscopy time of 5.2 ± 3.7 min (range: 0.3–18.3). Fluoroscopy duration showed a significant difference between groups based on AP localizations (*p* = 0.007). Advanced analysis revealed that the greatest difference was between the AS and PS locations (respectively, 0.18 ± 0.9 and 1.03 ± 2.8; *p* = 0.004). The procedure duration averaged 154 ± 54 min, with no significant difference between groups based on AP locations (*p* = 0.179). Furthermore, cryoablation and RFA procedure durations were similar (respectively, 143 ± 42 min, 138.3 ± 30.5; *p* = 0.751).

### 3.3. Overview of Initial and Redo Procedures Based on AP Location

#### 3.3.1. Anteroseptal Accessory Pathways

##### Initial Procedure

In AS APs (86; 29.5%), the acute success rate was 94.1%. Transient success occurred in four cases before immediate AP conduction returned. One (1.1%) AP showed Mahaim-like decremental conduction. Cryoablation, used in 94.2% of AS APs, had an acute success rate of 89.1%, with an average of 6.3 ± 3.1 complete lesions. Transient AV block occurred in 9.3% of patients, but they fully recovered. Six (6.9%) patients had recurrence of AP conduction. The mean procedure time was 153.5 ± 55.8 min.

##### Redo Procedure

Five (5.9%) patients underwent a second procedure, an average of 18.5 months after the initial ablation, with an average of 1.05 procedures per patient. All redo ablations were performed with cryoablation, with 100% acute success. In two patients, 8 mm-tip cryoablation catheters were preferred.

#### 3.3.2. Midseptal Accessory Pathways

##### Initial Procedure

Forty-six (15.9%) APs were in the MS location, with one (2.1%) exhibiting Mahaim-like properties. Transient success occurred in three cases before AP conduction returned, and two (4.3%) could not be eliminated. The acute success rate was 95.3%. Cryoablation, used in 81.3% of cases, had the highest efficacy (91.1% success). The average number of complete lesions was 6.9 ± 3.3. AP recurred in four (9.1%) cases. Mean procedure time was 144.6 ± 59.7 min.

##### Redo Procedure

A redo procedure was performed on six (13.9%) patients, an average of 17 months after the initial ablation, with a mean of 1.13 procedures per patient. Of the redo ablations, 50% were performed with cryoablation, whereas 33% were performed with RFA. In five patients, 8 mm-tip cryoablation catheters were preferred. No complication was seen during energy delivery. The long-term success rate was 97.6% with repeat procedures.

#### 3.3.3. Posteroseptal Accessory Pathways

##### Initial Procedure

PS APs (159; 54.6%) had the highest number of non-successful applications (14.4%) and longest procedure time (157.5 ± 51.8 min). CS ablations were performed in 32 patients (11.1%) with a 53.1% success rate. Cryoablation, used in eight cases, had the lowest efficacy in PS locations (80.7%) and the highest recurrence rate (18.9%). Overall, PS APs had a 14.4% recurrence rate. Three (1.8%) right-PS APs showed Mahaim-like properties. Recurrence rates were 16.4% for right-PS and 6.4% for left-PS APs, with no significant difference (*p* = 0.253). A transseptal puncture was required in 22 cases (7.6%) for left-PS AP localization.

##### Redo Procedure

A redo procedure was performed on 34 patients (21.3%), an average of 17.5 months after the initial ablation, with a mean of 1.21 procedures per patient. RF energy was prefered in 81.2% of the redo procedures, of which 60.6% used irrigated RF catheters. During the redo procedures, a combination of steerable long sheath (Agilis™, Abbott, Chicago, IL, USA) (12; 27.2%) and RF energy increased catheter stability and contributed to achieving successful outcomes.

### 3.4. Recurrence After a Second Procedure and Multiple (≥3) Procedures

Only three (1%) patients underwent ≥ 3 procedures for the initial substrate that recurred multiple times. Consequently, the long-term success rate reached 99% when considering the repeat procedures. 

**Figure 2:**
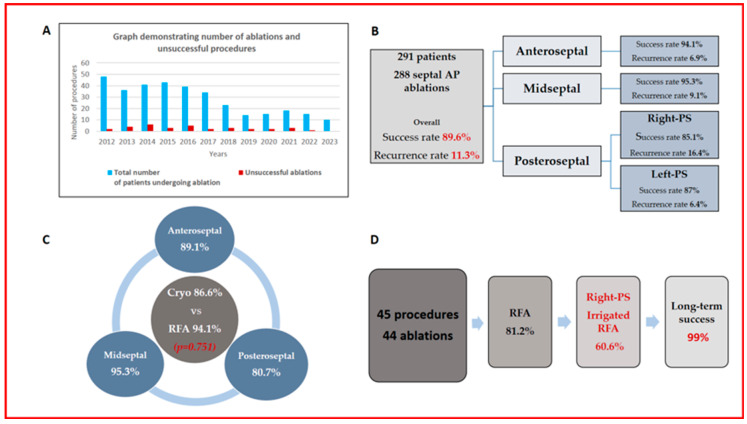
(**A**) The number of cases performed annually (blue bars) and the number of unsuccessful cases (red bars). (**B**) Accessory pathway and initial procedure characteristics of 291 patients. (**C**) Comparison of acute success rates between cryoablation and RFA, as well as cryoablation efficacy across different septal locations. (**D**) Overview of redo procedures and long-term success rates.

The summary of our study, encompassing the annual number of cases performed, characteristics of accessory pathways, and procedural details, is presented in Figure 2.

### 3.5. Complications

Transient complete AV block (4.8%) occurred during cryoablation, but patients fully recovered after tissue warming. Long-term follow-up showed no permanent PR prolongation or major complications, including pericardial effusion, thrombosis, or new-onset ischemia.

## 4. Discussion

This study supports contemporary pediatric data indicating that septal AP ablation can be performed safely, with a long-term success rate exceeding 98%. We observed a moderate recurrence rate with high long-term success, achieved with an average of 1.2 procedures per patient. Manifest APs and right-PS APs presented with the highest recurrence rate in the long term. RFA shows higher acute and long-term efficacy than cryoablation for septal AP, though the difference is not statistically significant. Cryoablation of PS APs showed the lowest acute and long-term efficacy rates compared to other septal APs. The use of cryotherapy and PS location were associated with an increased risk of requiring a repeat ablation. Furthermore, based on our experience, irrigated-tip catheters are effective and safe for right-PS APs resistant to conventional RFA or for those presenting with recurrences.

Cryoablation has emerged as a safer alternative catheter for septal APs, although the stiffness of the catheter makes manipulation more challenging in relatively small hearts [2,15]. Studies involving pediatric and adult populations have reported similar acute success rates for cryoablation and RFA, albeit with higher recurrence rates for cryoablation [5,7]. Ergul et. al. reported that the acute success rate of cryoablation for eliminating AS APs was 95.8%, with a short-term recurrence rate of 8.7% [16].

The first meta-analysis of 4244 pediatric studies found similar acute success rates for cryoablation and RFA, though efficacy depended on AP location [17]. Despite their similar acute efficacy rates, the meta-analysis demonstrates the superiority of RFA over cryoablation regarding long-term efficacy outcomes. In a study where Walsh et al. shared their 20 years of experience, they demonstrated that the use of cryotherapy is associated with increased risk of an unsuccessful cases and repeat procedures [18]. Cryoablation’s long-term efficacy was comparable to RFA for parahissian APs but lower at other septal sites. Our center previously reported a 93% acute success rate and 12.5% recurrence for cryoablation [19]. In our study, RFA and cryoablation showed excellent acute outcomes with no significant difference, though cryotherapy carried a higher risk of unsuccessful cases and repeat procedures. Success was highest in MS and lowest in PS locations, with our data supporting cryoablation’s long-term efficacy.

The PS region is the most complex area for catheter ablation, with higher recurrence rates. Epicardial APs often cause failed endocardial ablations, sometimes requiring CS intervention [20,21]. Studies suggest considering CS diverticula in failed cases and recommend coronary vein angiography to improve success [15,20,21,22,23,24]. Due to cryoenergy’s low success and high recurrence rates, data indicated that RFA was predominantly used in adult studies with better outcomes [22,23]. Our findings align with previous studies, showing the highest failure rates in CS ablations, possibly due to undetected coronary diverticula, as routine venography was not performed.

Coronary artery injury represents the most significant concern associated with intra-CS ablation with RF energy [15]. In a comprehensive study, coronary damage was observed in patients whose ablation area was within 5 mm of the coronary artery. No coronary artery injury was reported in patients with a true safety margin [25]. Although we did not perform coronary artery angiography before the procedure, all patients were closely monitored for ST changes for ischemia throughout the procedure. None of the patients developed clinical or ECG signs of new-onset ischemia.

In addition to the epicardial location in the PS area, the causes of recurrence were likely multiple, including insufficient lesion depth and inadequate energy delivery. Over the years, studies have indicated that irrigated RF energy is more effective on deep myocardial or epicardial substrates than on superficial endocardial structures [26,27]. Studies have reported improved procedural success rates and have proposed irrigated RF usage in particular as a strategy in redo procedures due to failure or recurrence [15,28]. In our study, the overall acute success rate for right-PS APs was 85.1%. Furthermore, right-PS APs exhibited the highest long-term recurrence rate (16.4%). We demonstrated that PS location predicts unsuccessful and repeat procedures, with cryoablation showing the lowest acute efficacy for PS AP. Consequently, we primarily utilized irrigated RFA in 60.6% of PS redo procedures. Given the numerous heat-related complications, including perforation, coronary damage, and the risk of venous stenosis in CS, we administered irrigated RF energy in a temperature-controlled mode ranging from 15 to 25 W within the CS and 25 to 35 W in the PS area. We attribute the low complication rate to these measures.

Despite technological improvements, extensive RFA experience, and cautious RF applications, the iatrogenic incidence of undesired AV block during septal ablations is still non-negligible. No case of persistent AV block was reported using cryoablation, whereas RFA accounts for between 2 and 10% [4,17,29]. Knowledge of these risks may influence both the patients’ and the electrophysiologists’ decisions. Ergul et al. reported that RFA was not performed in 29% of their patients who underwent previous EPSs at other centers due to the high risk of AV block [16]. Thus, despite the concerns regarding a greater recurrence rate, cryoenergy has been recommended for AS and MS locations due to AV block risk reported for RFA [4,5,6,7,8,29]. In our study, only cryoablation was preferred in 94.1% of AS and 76% of MS APs. There were no instances of permanent damage to normal conduction in our series. In addition to monitoring AV node conduction closely during mapping and ablation, we emphasize confirmation of underlying intact AV node conduction with differential atrial pacing maneuvers before and during the ablation. Using long sheaths, a superior approach via the jugular vein in selected patients, and apnea during energy delivery may also increase catheter stability and help limit the risk of AV node injury.

Previous studies have reported that Ebstein’s anomaly poses a risk for failed ablation and recurrence, with difficulties being related to the high prevalence of multiple pathways and the lack of catheter stability caused by the displacement of the tricuspid valve [15,18,30]. In our study, we did not observe procedural failure or recurrence in patients diagnosed with Ebstein’s anomaly. However, we believe that the limited number of patients in our study is insufficient to draw such a conclusion.

The use of 3D mapping systems during electrophysiological studies in pediatric patients plays a crucial role in procedural outcomes by providing precise visualization and guidance. Employing an electroanatomic mapping system aids in targeting locations and helps place the insurance lesions at precise anatomical locations. Additionally, reducing X-ray exposure is particularly important for patients requiring multiple procedures, as cumulative radiation exposure poses potential long-term risks.

We have achieved an excellent long-term success rate. It can be argued that reversible tissue injury represents a relative weakness of cryoablation concerning recurrence risk. However, this aspect may also be a major strength of the technology when ablating near-normal conduction tissues. In our opinion, the many advantages of general anesthesia like catheter stability, lack of patient movement, and better tolerance for longer procedures; larger catheter tip size; and a greater number of cryoenergy applications may have affected our long-term success rate. This is because the freeze–thaw–freeze technique has been reported to result in more effective lesion formation. With RFA, balancing lesion consolidation against AV block and coronary injury risk is key, making limited consolidation time a reasonable recurrence. Using steerable sheaths, irrigated RF catheters, and angiograms may further improve outcomes.

## 5. Study Limitations

While this study provides a comprehensive overview of pediatric AP ablation outcomes, several limitations exist. As this is a retrospective review, selection bias is possible. Variability in catheter types, energy settings, operator experience, and techniques could affect outcomes and recurrence rates. The absence of coronary sinus injection before CS ablations may contribute to lower acute success, and challenges in establishing symptom–rhythm correlation in children might underestimate recurrences. Additionally, certain underlying factors that may contribute to procedural failures and recurrences are beyond the scope of the study.

## 6. Conclusions

Pediatric ablations are very safe, with the risk of complete heart block requiring a pacemaker and other major complications being extremely low (<0.01%). Despite impressive acute success rates, recurrences remain higher in septal AP ablations compared to other locations. Therefore, repeated procedures may be necessary to achieve definitive long-term success. The results of this study indicate that comparable acute and long-term success rates can be achieved with cryoablation compared to RF ablation, along with the significant added benefit of increased safety with cryoablation in this patient group.

## Figures and Tables

**Table 1:** Demographic and clinical characteristics of 291 patients.

Characteristics	
Age (years)	11.8 ± 4.9
Gender (M/F)	171/120
Weight (kg)	48.8 ± 27.3
Echocardiogram findings	
VSD	2
Ebstein’s anomaly	7
TOF	2
D-TGA	1
CAVSD	1
Dextrcardia/Tricuspit atresia/DOLV/PB	1
Bicuspit aorta/Aortic insufficiency	4
HCM	2
DCM	1
Indications of procedure (ECG findings)	
SVT	79 (27.1%)
WPW pattern (asymptomatic)	71 (24.5%)
WPW syndrome	
WPW–SVT (AVRT)	140 (48.1%)
Pre-excited AF	1 (0.3%)
Prior EPS (at another center)	10 (3.4%)
Follow-up time (months)	88.5 ± 33.0

F = female; M = male; VSD = ventricular septal defect; TOF = tetralogy of Fallot; D-TGA = D- transposition of the great arteries; CAVSD = complete atrioventricular septal defect; DOLV = double-outlet left ventricle; PB = pulmonary banding; HCM = hypertrophic cardiomyopathy; DCM = dilated cardiomyopathy; WPW = Wolff–Parkinson–White; AF = atrial fibrillation; SVT = supraventricular tachycardia; EPS = electrophysiological study; ECG = electrocardiogram.

**Table 2:** Pathway and initial procedure characteristics of 291 patients.

Characteristics	n (%)
Multiple AP *	7 (2.4%)
AP characteristics (n)	298
Manifest AP	225 (75.5%)
Concealed AP	71 (23.8%)
PJRT	2 (0.7%)
Risk classification of AP	225
APERP, SPERRI, and SPPCL ≤ 250 msn	61 (28%)
APERP, SPERRI, and SPPCL = 251–300 msn	77 (34.5%)
APERP, SPERRI, and SPPCL ≥ 300 msn	82 (36.5%)
Unknown	5 (1%)
APERP	294.4 ± 5.3
Adenosine-responsive	90 (40%)
Septal AP locations	291
Anteroseptal	86 (29.5%)
Midseptal	46 (15.9%)
Posteroseptal	159
Right	128 (43.9%)
Left	31 (10.7%)
Epicardial ablation	32 (11.1%)
Second arrhythmia substrate (AVNRT)	22 (7.5%)
Use of fluoroscopy (n)	39 (13.4%)
Transseptal puncture	22
Other reasons	17
Overall fluoroscopy time (min)	5.2 ± 3.7 (0.3–18.3)
Overall procedure time (min)	154 ± 54 (54–340)

* 3 left lateral, 1 left posterior, 2 right posterior, 1 right lateral location. AP = accessory pathway; WPW = Wolff–Parkinson–White; PJRT = permanent junctional reciprocating tachycardia; APERP = effective refractory period of the accessory pathway; SPERRI = shortest pre-excitatory RR interval; SPPCL = shortest paced cycle length with pre-excitation during rapid atrial pacing; AVNRT = atrioventricular nodal re-entrant tachycardia.

**Table 3:** AP location and ablation outcomes.

SEPTAL APs				
	Anteroseptal	Midseptal	Posteroseptal	
	(*n*)	(*n*)	(*n*)	
**Initial Procedures**			**Right-PS**	**Left-PS**
EPS (291; %)	86 (29.6)	46 (15.8)	128 (43.9)	31(10.7)
Ablations (288; %)	86(29.9)	43 (14.9)	128 (44.5)	31(10.7)
Ablation source (n; %)				
Cryo (190; 66%)	81(94.1)	35 (81.4)	68(53.1)	6 (19.3)
RF (30; 10.4%)	0	2 (4.7)	10 (7.8)	18 (58)
Cryo + RF (62; 21.5%)	5 (5.8)	6 (13.9)	46 (35.9)	5 (16.1)
Irrigated tip RF (6; 2%)	0	0	4 (3.1)	2 (6.4)
Transient complete AV block (4.8%)	8 (9.3)	3 (6.9)	3 (2.3)	0
Acute success rate (89.6%)	81 (94.1%)	41 (95.3%)	109 (85.1%)	27 (87.0%)
Recurrence rate (11.3%)	6 (6.9%)	4 (9.1%)	21 (16.4%)	2 (6.4%)
**REDO PROCEDURES** **(Initial procedure failure + recurrences)**				
EPS (45; %)	5 (11.1)	6 (13.3)	31 (68.8)	3 (6.6)
Ablations (44; %)	5 (11.3)	6 (13.6)	30 (68.1)	3 (6.8)
Ablation source (n; %)				
Cryo (13; 29.5%)	5 (100)	3 (50)	5 (15.1)	0
RF (9; 20.4%)	0	2 (33.3)	5 (16.6)	2 (66.6)
Cryo + RF (2; 4.5%)	0	1 (16.6)	1 (3.3)	0
Irrigated RF (20; 45.4%)	0	0	19 (63.3)	1 (33.3)
Transient complete AV block (4.6%)	2 (40)	0	0	0
Acute success rate (95.3%)	5 (100%)	5 (83.3%)	28 (93.3%)	3 (100%)
Long-term success rate (99%)	100%	97.6%	98.4%	100%

AP = accessory pathway; EPS = electrophysiological study; Cryo = cryoablation; RF = radiofrequency, AV = atrioventricular block.

**Table 4:** Acute and long-term outcomes according to location of accessory pathway.

	Anteroseptal	Midseptal	Posteroseptal	*p* ^1^	*p* ^2^	*p* ^3^	*p* ^4^
**Initial procedure acute success**	81 (94.1%)	41 (95.3%)	136 (85.5%)	0.110	0.056	0.02 *	0.232
**Initial procedure recurrence ^a^**	6 (6.9%)	4 (9.1%)	23 (14.4%)	0.171	0.083	0.308	0.739
**Long-term success**	86/86 (100%)	45/46 (97.6%)	157/159 (98.7%)	0.356	0.861	0.762	0.873

Values are expressed as no./no. (%). ^a^ Substrates with a redo procedure that were re-treated were included. *p*^1^, overall; *p*^2^, AS vs. PS; *p*^3^, MS vs. PS; *p*^4^, AS vs. MS. * Statistically significant.

**Table 5:** Risk factors for requiring a repeat ablation for accessory pathways.

Factor	OR	95% CI
Reference	1.0	-
Female Gender *	2.2	(1.097–4.386)
Age < 8	2.1	(0.642–6.860)
Weight < 30 kg	1.8	(0.579–5.696)
Cryo Catheter *	10.7	(1.008–113.365)
Posteroseptal AP *	3.2	(1.168–8.972)

* Significant with *p* < 0.05. AP = accessory pathway; CI = confidence interval; OR = odds ratio.

## Data Availability

Data are not publicly accessible due to patient privacy considerations; however, they can be obtained from the corresponding author upon reasonable request.

## Abbreviations

Aps	Accessory pathways
WPW	Wolff–Parkinson–White
RFA	Radiofrequency ablation
